# Authorization of COVID-19 clinical trials: lessons from 2 years of experience of a national competent authority

**DOI:** 10.3389/fphar.2022.972660

**Published:** 2022-08-15

**Authors:** Stéphane Vignot, Alban Dhanani, Isabelle Sainte-Marie, Laure de Ligniville Lajavardi, Gwennaelle Even, Muriel Echemann, Nina Hulin, Claire Ménoret, Patrick Maison, Christelle Ratignier-Carbonneil

**Affiliations:** Agence Nationale de Sécurité du Médicament et des Produits de Santé (ANSM), Paris, France

**Keywords:** regulatory science, clinical trials, COVID-19, national competent authorities, repurposing strategies

## Abstract

The COVID-19 pandemic was immediately marked by strong clinical research activity. The French national competent authority presents the data on request for authorization during the first 2 years of COVID-19 pandemic to inform discussions on future clinical research issues. Applications for authorization of interventional COVID-19 trials submitted between March 2020 and February 2022 were analysed. Trials on medicinal products were classified according to market authorization status, mechanism of action of the investigational product, target population and clinical context. In 2 years, 208 clinical trials were submitted. 75% were authorized, 3% refused, 22% withdrawn by the sponsor. Among medicinal products trials, 6% were adaptative, 28% included outpatients and 2% were focused on post COVID-19 symptoms. Vaccines were evaluated in 9% of trials, antivirals in 38% and immunomodulators in 35%; 63% of antiviral and 60% of immunomodulation trials included a drug with a marketing authorization in another indication. The dynamics of authorization prove the involvement of stakeholders but also illustrates the risk of dispersion of research efforts and the risk of decorrelation between trials and the epidemic evolution. The high rate of withdrawal of applications could be explained by changes in the sanitary context and by the dropping of some therapeutic approaches. Most of clinical trials evaluate drugs authorized in another indication and assessment procedures by authorities have to mitigate between the knowledge of safety profile of those drugs and the uncertainty in a new clinical context with rapidly evolving knowledge. COVID-19 experience should now support future evolution in clinical research practices.

## Introduction

During the emergence of the COVID-19 pandemic, from March 2020 in France, the national competent authorities (NCA) have faced a double issue in the field of clinical trials: adapt the monitoring of ongoing clinical trials and enable the rapid implementation of dedicated trials to define evidence-based management. To address the first objective, the ANSM (Agence Nationale de Sécurité du Médicament et des Produits de Santé) has proposed national guidelines in line with the European recommendations (Vignot et al., 2020; EC.Europa, 2021). Concerning the second aspect, we propose in this article to present the dynamics of submission of COVID-19 trials to the ANSM as well as the typology of trials and applicants. The proposed types of drugs and target populations can be detailed over the whole period and by semester to discuss the correlation between the evolution of research strategies and the evolution of the pandemic. This work is intended to provide transparency and to discuss the lessons that can be learnt about the conduct of clinical trials in the context of a health crisis, but also for the optimisation of clinical research in Europe. The presentation of authorization data also provides an opportunity to outline a competent authority’s approach for clinical trials assessment in a context of maximum initial uncertainty in a pandemic situation.

## Materials and methods

Applications for initial approval of clinical trials for the management of SARS-CoV-2 infection, its complications or its prevention submitted between 1 March 2020 and 28 February 2022 were considered. All studies subjected to authorization by the ANSM were included in the analysis concerning drugs, plasma use, medical devices and risky interventional trials (so-called “non-health product” trials) requiring authorization by the competent authority in French regulation.

Non-interventional or low interventional studies for which no authorization from ANSM is required were not considered. Post-vaccination follow-up cohorts or post-treatment studies with monoclonal antibodies specific to SARS-CoV-2 were subjected to a specific derogatory regime not requiring authorization by the competent authority even in the case of an additional follow-up procedure.

The epidemiological data considered in relation to the dynamics of clinical trial submission are extracted from French public data (Santé Publique France) (Santé Publique France, 2020).

## Results

### Clinical trials submitted to the ANSM between March 2020 and February 2022

**FIGURE 1:**
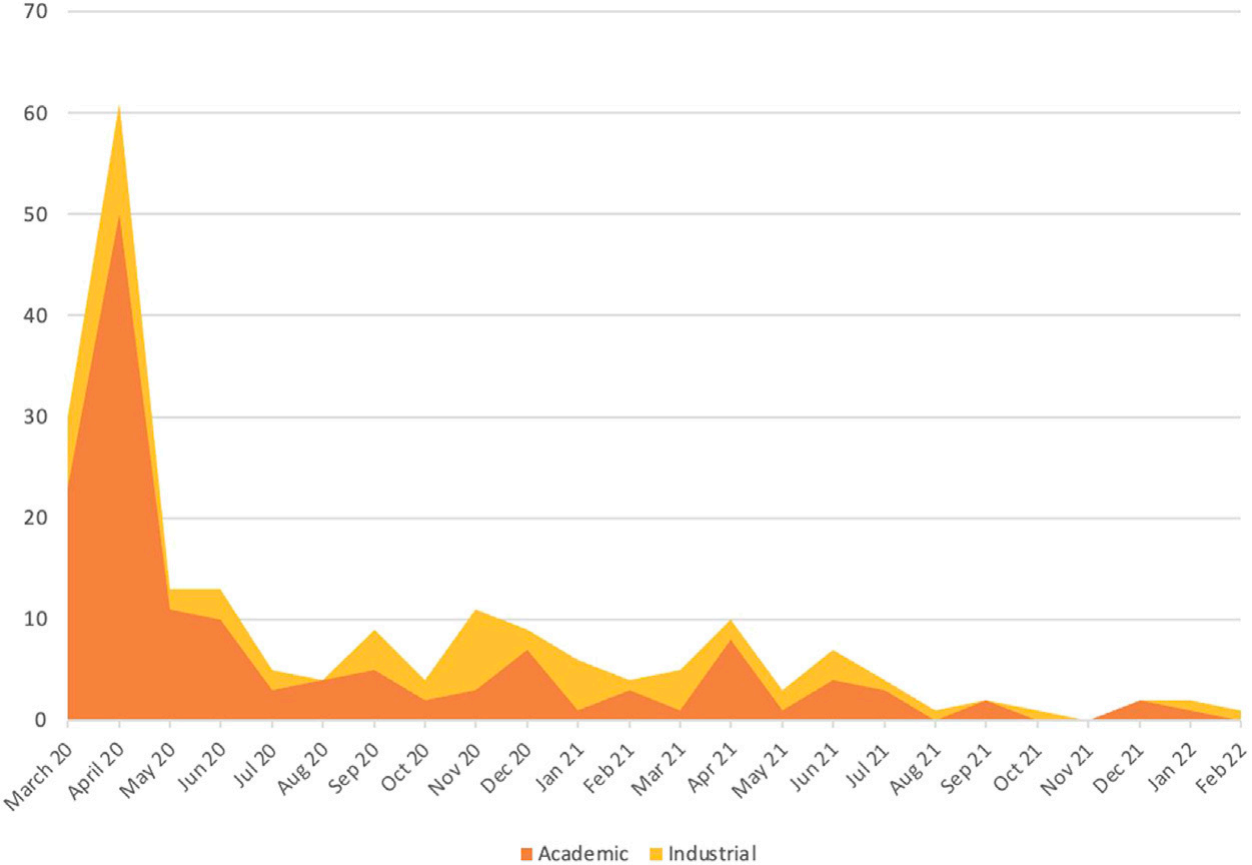
Submission rate in France according to the type of sponsor (March 2020 - February 2022).

A total of 208 interventional clinical trials related to SARS-CoV-2 infection were submitted between March 2020 and February 2022, representing 8.4% of the 2,463 applications submitted to ANSM during this period. The majority (84%) of COVID-19 related trials investigates medicinal products (Table 1). The submission rate is shown in Figure 1 with stratification according to the type of sponsor (academic or industrial). Over the entire period under consideration, trials with academic sponsorship represent 69% of the dossiers submitted to the ANSM.

**TABLE 1:** COVID-19 interventional clinical trials submitted in France (March 2020 - February 2022).

	Submitted	Authorized
Medicinal product	176	126
Convalescent plasma	2	2
Medical device	4	4
Interventional Clinical Trial without health product	26	24
Total	**208**	**156**

**FIGURE 2:**
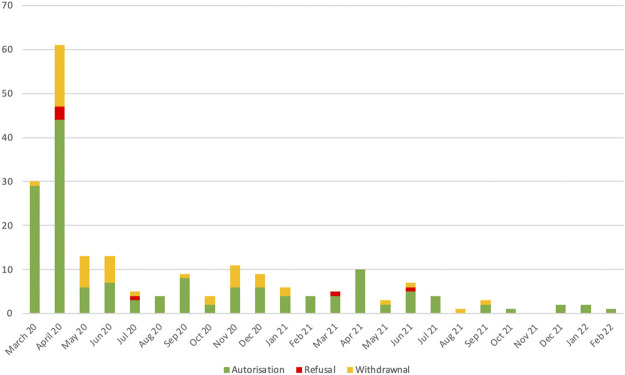
Decision on COVID-19 interventional clinical trials submitted in France.

Of the trials submitted, 75% were authorized (156 trials), 3% were refused (6 trials), 22% were withdrawn by the sponsor during the initial evaluation of the dossier (46 trials). Details by time period are shown in Figure 2.

A focus on the first epidemic wave (March 2020 to May 2020) is proposed in Figure 3, with correlation between the kinetics of submission by the sponsor, decision by the NCA and the epidemic evolution in France. Trials submitted during the first wave represent 50% of the total number of COVID-19 trials submitted (104 out of 208) and 47% of authorized trials (74 out of 156).

### Authorized medicinal product trials

**FIGURE 3:**
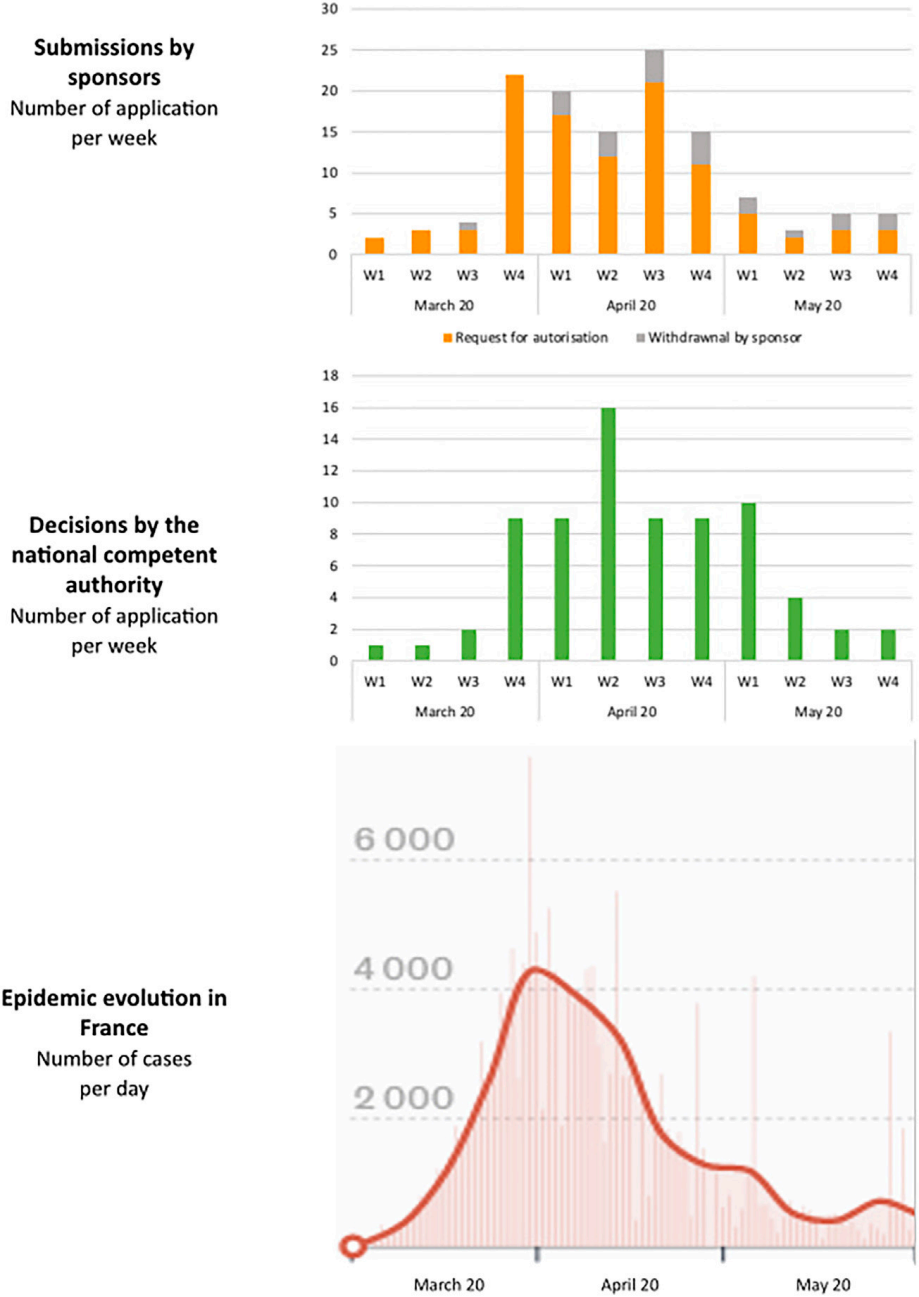
Focus on the first epidemic wave (March 2020 to May 2020). Correlation between submissions by sponsors, decisions by the national competent authority and the epidemic evolution in France.

#### Investigated strategies

128 clinical trials on medicinal products were authorized during the considered period. The target populations and management contexts evaluated are presented in Table 2 (global view and by semester). Adaptive platform trials, that include the possibility of evaluating several strategies, simultaneously or by iteratively adding new ones, represent 6% of trials. National trials were predominant during the first year (70%) and thereafter the proportion was reversed in favour of international trials (62% between March 2021 and February 2022).

**TABLE 2:** Description of authorized COVID-19 clinical trials on medicinal products (global view and by semester).

		Total		March 20 August 20		Sept 20 February 21		March 21 August 21		Sept 21 February 22	
			%	N	%	N	%	N	%	N	%
Number of trials		128	100%	74	100%	25	100%	23	100%	6	100%
Adaptative platform trials		8	6%	6	8%	0	0%	1	4%	1	17%
International trials		48	38%	22	30%	8	32%	14	61%	4	67%
Target population											
COVID- subjects (healthy or at risk of complications)		19	15%	5	7%	5	20%	8	35%	1	17%
Outpatients		16	13%	5	7%	7	28%	4	17%	0	0%
Non severe inpatients		42	33%	27	36%	7	28%	6	26%	2	33%
Moderate to severe and severe inpatients		51	40%	37	50%	6	24%	5	22%	3	50%
Contexte											
Prevention of infection		19	15%	5	7%	5	20%	8	35%	1	17%
Vaccines		12	9%	1	1%	3	12%	7	30%	1	17%
Pre-exposure treatment		6	5%	4	5%	2	8%	0	0%	0	0%
Post-exposure treatment		1	1%	0	0%	0	0%	1	4%	0	0%
COVID specific treatments		94	73%	62	84%	14	56%	13	57%	5	83%
Intensive care treatments		8	6%	5	7%	3	12%	0	0%	0	0%
Anticoagulation for COVID patients		5	4%	2	3%	3	12%	0	0%	0	0%
Sequelae after COVID		3	2%	1	1%	0	0%	2	9%	0	0%

Trials initially focused on inpatients (86% of trials between March and August 2020). Between September 2020 and August 2020, the proportion between outpatient and inpatient trials was balanced (50% each). Inpatient trials went back to the majority share after September 2021 (83%). In total, outpatient trials account for 28% of the applications.

#### Drugs being investigated: vaccines, antiviral strategies, immunomodulation, repositioning in clinical trials

The types of drugs (including cell therapy, plasma and immunoglobulins) that were tested are shown in Table 3.

**TABLE 3:** Drugs being investigated in COVID-19 clinical trials on medicinal products (global view and by semester).

		Total		March 20 August 20		Sept 20 February 21		March 21 August 21		Sept 21 February 22	
		**N**	**%**	N	%	N	%	N	%	N	%
Number of trials		**128**	**100%**	74	119%	25	100%	23	100%	6	100%
COVID-19 vaccines		**12**	**9%**	1	2%	3	12%	7	30%	1	20%
Antiviral drugs		**49**	**38%**	28	45%	12	48%	6	26%	3	60%
SARS-CoV2 specific antibodies		**5**	**4%**	1	2%	1	4%	3	13%	1	20%
Hydroxychloroquine		**14**	**11%**	14	23%	0	0%	0	0%	0	0%
Renin Angiotensin System		**8**	**6%**	6	10%	2	8%	0	0%	0	0%
Other		**22**	**17%**	7	11%	9	36%	3	13%	2	40%
Immunomodulative drugs		**45**	**35%**	34	55%	2	8%	7	30%	2	40%
Corticosteroids		**8**	**6%**	7	11%	1	4%	0	0%	0	0%
Anti IL6 Mab		**5**	**4%**	4	6%	0	0%	0	0%	1	20%
Anti IL1 Mab		**4**	**3%**	4	6%	0	0%	0	0%	0	0%
JAK inhibitor		**3**	**2%**	3	5%	0	0%	0	0%	0	0%
Other		**25**	**20%**	16	26%	1	4%	7	30%	1	20%
Plasma and Human immunoglobulines		**6**	**5%**	4	6%	1	4%	1	4%	0	0%
Cellular therapies		**3**	**2%**	2	3%	0	0%	0	0%	1	20%
Other (supportive or intensive care therapies, anticoagulation)		**13**	**10%**	5	8%	7	28%	2	9%	0	0%

Vaccine trials represent 9% of the authorized on medicinal product trials, most between September 2020 and August 2021 (10/12). Besides vaccines, clinical drug trials focused on two main approaches: antiviral strategies (38% of drug trials) and immunomodulation strategies (35% of drug trials). Among these trials, repurposing of already known molecules was considered. A distinction is made between molecules that already have marketing authorization (63% of antiviral trials, 60% of immunomodulation trials) and molecules under development in another indication without marketing authorization (24% of antiviral trials, 38% of immunomodulation trials). Molnupinavir was considered as a repurposing drug since the compound was originally developed for use against another virus (Hampton, 2020). Finally, 10% of antiviral trials evaluated drugs developed specifically for the treatment of SARS-CoV-2 (Table 4).

**TABLE 4:** Repurposing strategies for antiviral and immunomodulation drugs in COVID-19 clinical trials (global view and by semester).

		Total		March 20 August 20		Sept 20 February 21		March 21 August 21		Sept 21 February 22	
		**N**	**%**	N	%	N	%	N	%	N	%
Antiviral drugs		**49**		28		12		6		3	
Specific		**5**	**10%**	1	4%	1	8%	1	17%	2	67%
Repurposing with MA		**31**	**63%**	22	79%	8	67%	1	17%	1	33%
Repurposing without MA		**12**	**24%**	5	18%	3	25%	4	67%	0	0%
Immunomodulative drugs		**45**		34		2		7		2	
Repurposing with MA		**28**	**60%**	24	71%	2	100%	0	0%	2	100%
Repurposing without MA		**17**	**38%**	10	29%	0	0%	7	100%	0	0%

## Discussion

Clinical trials for the management of SARS-CoV-2 infection, its complications or its prevention are registered on public databases by the sponsors (www.clinicaltrials.gov) or by the competent authorities of the EU Member States (www.clinicaltrialsregister.eu). These registers provide transparency on authorized trials but do not include all information about applications for authorization submitted to the competent authorities. Trials whose authorization has been refused or whose application has been withdrawn by the sponsor during the initial assessment are not systematically reported. The presentation of all trials submitted in France during the first 2 years of the pandemic, between 1 March 2020 and 28 February 2022, aims to reinforce the information on COVID-19 clinical research.

The submission kinetics of clinical trials show a rapid involvement of sponsors in France, in particular from academia, from the very first weeks of the pandemic. The submission dynamic was accompanied by the responsiveness of the competent authorities (median evaluation time of less than 7 days for both ANSM and the ethics committees in March and April 2020). Comparison with the rate of contamination during the first wave shows, however, that the maximum number of clinical trial submissions and authorizations was achieved at a time when the maximum level of contamination had already been reached. Although sponsors and institutions sought to deploy clinical trials as quickly as possible after regulatory authorization, the maximum offer of clinical research could only be proposed in the latter part of the first wave, as France experienced a first early epidemic peak in March 2020 followed by a rapid decrease in the number of cases.

A large number of clinical trials were active at the end of May 2020 (79 trials), while the number of infections had decreased and the risk of a subsequent resurgence of the epidemic was not known at that time. The major contrast between the initial dynamism of the clinical research actors and the final observation of a number of studies disproportionate to the epidemic situation in summer 2020 led to questioning the risk of dispersion of the clinical research forces and underlined the need to rapidly identify research priorities (Sipido et al., 2020). In France, a national priority label has been proposed to provide institutional support for trials likely to provide the most effective scientific answers to the challenges of managing SARS-CoV-2 infection and its complications, considering both scientific quality and implementation capabilities in the national health context. The label is granted following an independent scientific evaluation under the aegis of the Ministries of Health and Research, with the participation of ANRS-MIE (*Agence Nationale de Recherche sur le SIDA - Maladies infectieuses émergentes*), the national agency created to monitor emerging infectious diseases (Telford et al., 2021). This process must be distinguished from the authorization of the ANSM, the competent authority, and the opinion of the ethics committees. It is not intended to create an additional step before authorization of a clinical trial. The application for a national priority label is optional, at the discretion of the sponsor if it wishes to have institutional support and greater visibility. The application for authorization will be assessed according to the same criteria by the authorities (national competent authority and ethics committee), whether or not there is an application for a label, and whether or not the label has been obtained, in order to guarantee the safety of the patients, the respect of their rights and taking into account ethical considerations. As illustrated in Figure 2, the submission rate during the subsequent epidemic peaks was lower, which leads us to consider the proposed national label as a tool to help research in infectious diseases, but does not allow us to evaluate its impact in a situation of dispersed research efforts such as that observed in March and April 2020.

The overall vision of the profile of COVID-19 trials may lead to question the adequacy between the research objectives and the evolution of the pandemic. The proportion and timing of vaccine trials is consistent with the health situation and the progress of national vaccination coverage. In France, the national vaccination campaign was initiated at the end of 2020, initially targeting healthcare workers and at-risk populations, and then opened to the entire population in May 2021. Outside vaccine trials, the evolution of target populations allows us to identify 3 phases: an initial predominance of inpatient trials, a rebalancing between inpatient and outpatient trials, and then a re-increase in the proportion of inpatient trials (with a smaller number of trials). This may be explained by the evolution of the pandemic and by operational considerations. In the first period, the urgent unmet medical needs were focused on the surge of severe patients in hospitals and on the burden in intensive care or reanimation units. The improvement of clinical management practices (diagnosis, ventilation, monitoring), the reduction of the epidemic pressure and the use of corticosteroids and immunomodulators (tocilizumab, anakinra) have reduced the hospital pressure (Cammarota et al., 2021; Khan et al., 2021; The WHO Rapid Evidence Appraisal, 2021; Wagner et al., 2021; Faz zini et al., 2022). At the same time, better knowledge of the history of the disease has highlighted the importance of early treatment, particularly for antiviral strategies, with priority given to patients at risk of complications. This situation has consequently become an important urgent medical need for which the emergence of effective therapeutic strategies has been successful: vaccination, of course, in the first instance, but also authorization of compassionate access in France to monoclonal antibodies since February 2021 (ANSM, 2020) and subsequently to the antiviral nirmatrelvir/ritonavir since January 2022 (ANSM, 2021). The availability of these different therapeutic alternatives outside of clinical trials may have led to a re-focusing of the need for new therapies for hospital patients. At the same time, however, operational considerations should not be ignored. Only hospitals had sufficient experience of clinical research and trained staff to allow the rapid implementation of clinical trials when the pandemic emerged. There was also a strong willingness to set up outpatient trials, involving community practitioners, hospital structures, academic or industrial sponsors and the authorities (Ministry of Health, ANRS-MIE, ANSM). All the stakeholders pointed out the difficulties in setting up and including ambulatory patients, underlining the importance of implementing specific training and support actions, beyond the epidemic situation. These difficulties may therefore also explain the progressive refocusing of research projects on hospitals. This observation should raise questions about the capacity to set up trials outside hospital structures and about the possibility of conducting ambulatory trials under optimal conditions of safety and reliability. Trials relating to post COVID management are few (2%) but it should be pointed out that non-interventional or low-interventional studies not involving drugs have been set up in parallel, not requiring authorization from the national competent authorities.

Concerning the procedures of assessment of clinical trials by the competent authority, a high rate of withdrawal of authorization requests has been observed (up to 30% of dossiers between May and November 2020 compared to less than 10% for COVID-19 dossiers submitted in 2021 and a withdrawal rate of 10% for clinical trials submitted in 2018 - public data from the ANSM) (ANSM, 2022). This high rate can be explained on the one hand by a mismatch between a research project and its actual feasibility according to the evolution of the health context (recruitment capacity not achievable during periods of epidemic decline, impact of vaccination on the risk of hospitalisation) and on the other hand because of the rapid evolution of knowledge that can invalidate a research question that was relevant at the time of the design of the protocol. Some sponsors may additionally have faced some difficulties in providing the appropriate elements to build their application dossier. This experience underlines the importance for authorities to be able to provide rapid scientific advices at both national and European level. The training of academic actors in regulatory science needs to be further strengthened (Starokozhko et al., 2021).

The examples of trials including a regimen with hydroxychloroquine or evaluating the place of corticosteroids can be mentioned. The first clinical results studying the use of hydroxychloroquine have led to the drop-out of this approach in clinical trials submitted in France after June 2020 (Boulware et al., 2020; Funnell et al., 2020; Molina et al., 2020; T ang et al., 2020); meanwhile, the increased knowledge on the place of corticosteroids in clinical management led to the modification of the research questions from July 2020 (Horby et al., 2021; Tomazini et al., 2020). The need to take into account the rapid and unprecedented evolution of knowledge was an issue shared by all stakeholders, including sponsors and regulatory authorities. The initial assessment of the clinical trial applications was conducted in a situation of maximum scientific uncertainty: lack of knowledge on the physiopathology and natural history of COVID-19, absence of a validated preclinical model, absence of consensual standard of care, urgent unmet medical need and context of maximum health pressure. Under these conditions, a regulatory authority must ensure that these uncertainties are taken into account and that there is no unacceptable excess risk for patients, that the conditions for monitoring patients are accurately described, and that the evolution of knowledge is taken into account to guarantee that patients can receive a new standard of treatment that may emerge.

In the context of the rapid emergence of a new pathogen, the drugs initially evaluated in clinical trials logically corresponded to drugs developed for other indications (repurposing strategy) (Namasivayam et al., 2022; Rodrigues et al., 2022). The compounds developed specifically against SARS-CoV-2 were introduced into clinical trials in a second time (first submission in France of a vaccine trial in June 2020, and of a monoclonal antibody trial in November 2020). For repurposing purposes, a distinction needs to be made between molecules that already have a marketing authorization and molecules under development in another indication without marketing authorization. Clinical experience is different and knowledge of drug safety is lower in the latter case than when used in a real-life setting. However, in both cases, it remained crucial not to consider that the knowledge of a safety profile in a given clinical context implies a perfect transposition to a new situation. The use of a known molecule in a new physiopathological context should not exempt close monitoring of safety and management of all toxicity risks within the trial. This point is a persistent challenge for repositioning trials, particularly but not exclusively for trials aimed at COVID-19 + patients who are at the highest risk of complications and potentially affected by comorbidities.

In conclusion, the 2-years review of the COVID-19 clinical trial authorization process highlights the intense involvement of stakeholders in rapidly proposing research protocols and evaluating different repurposing strategies. However, the goodwill shared by all concerned is not enough. The dispersion of research and the lack of adequacy with research priorities should make us wonder about the ways to enable better coordination and greater responsiveness in the research in health emergency situations. The development of adaptive platform trials is an option to be strengthened. These represented only 6% of the COVID-19 clinical trials authorized in France, even though they are able to answer important questions, both for the management of hospitalised patients and outpatients, and for vaccine strategies. Publications confirm the importance of this type of trials in international COVID-19 research. Platform trials offer flexibility and responsiveness with the possibility of adding patient groups and new treatments to an already active trial (i.e., active research sites, trained investigators). Regulatory authorities can rely on the experience gained over the past decade in the initial assessment of these trials, particularly in oncology. However, it is important to remind some risks to be taken into account. Sponsors should be aware that the use of adaptive designs should not compromise scientific consistency (Sudhop et al., 2019). The comparison between groups by period may raise concerns due to changes in variants and practices (the 2020 control arm is no longer the 2022 control arm) (Roth et al., 2021). Particular care should also be focused on the implementation of platform trials as they can become complex packages and their operational management becomes a source of confusion for both sponsors and regulatory authorities (national competent authorities and ethics committees). The risk of misunderstanding for patients and investigators must be taken into account as a matter of priority, especially in a context where the health situation may lead to tensions in routine care. The complexity of a clinical trial should not lead to a reduced capacity for inclusion and quality of follow-up of research participants.

## Data Availability

The datasets presented in this study can be found in online repositories. The names of the repository/repositories and accession number(s) can be found below: Data on clinical trials in EU are available on www.clinicaltrialsregister.eu.
